# Exploring the Evolution-Coupling Hypothesis: Do Enzymes’ Performance Gains Correlate with Increased Dissipation?

**DOI:** 10.3390/e27040365

**Published:** 2025-03-29

**Authors:** Davor Juretić

**Affiliations:** Faculty of Science, University of Split, Ruđera Boškovića 33, 21000 Split, Croatia; juretic@pmfst.hr or davor.juretic@gmail.com

**Keywords:** evolution, specialized enzymes, entropy production, kinetic constants, scaling laws, catalytic efficiency, turnover number, dissipation, generalist enzymes, stochastic noise

## Abstract

The research literature presents divergent opinions regarding the role of dissipation in living systems, with views ranging from it being useless to it being essential for driving life. The implications of universal thermodynamic evolution are often overlooked or considered controversial. A higher rate of entropy production indicates faster thermodynamic evolution. We calculated enzyme-associated dissipation under steady-state conditions using minimalistic models of enzyme kinetics when all microscopic rate constants are known. We found that dissipation is roughly proportional to the turnover number, and a log-log power-law relationship exists between dissipation and the catalytic efficiency of enzymes. “Perfect” specialized enzymes exhibit the highest dissipation levels and represent the pinnacle of biological evolution. The examples that we analyzed suggested two key points: (a) more evolved enzymes excel in free-energy dissipation, and (b) the proposed evolutionary trajectory from generalist to specialized enzymes should involve increased dissipation for the latter. Introducing stochastic noise in the kinetics of individual enzymes may lead to optimal performance parameters that exceed the observed values. Our findings indicate that biological evolution has opened new channels for dissipation through specialized enzymes. We also discuss the implications of our results concerning scaling laws and the seamless coupling between thermodynamic and biological evolution in living systems immersed in out-of-equilibrium environments.

## 1. Introduction

### 1.1. What Is the Relationship Between Universal (Thermodynamic) and Biological Evolution?

Evolution is predominantly associated with biological evolution, the most familiar type [1]. However, non-equilibrium spatiotemporal evolution is a powerful unifying concept across various scientific disciplines. Ludwig Boltzmann laid the groundwork for linking thermodynamics and biological evolution [2]. In 1922, Alfred Lotka argued for the idea [3] that is described today as the maximum entropy production principle as long as the store of the available energy is not exhausted [4,5]. Half a century after Boltzmann, the brilliant physicist Erwin Schrödinger introduced his famous notion that “life feeds on negative entropy” [6]. In present-day terminology, we would describe his insight as life’s ability to harvest high-free-energy entities (photons, organic compounds) from its environment and convert them into the proton-motive force and chemical affinities before exporting their dissipated remains (waste products and heat) to the environment. That general intuition later became quantitative, primarily due to the contributions of Terrell L. Hill [7], Hong Qian [8], and others, including the Nobel laureate Ilya Prigogine. The general evolution criterion [9] and the universal evolution criterion for nonlinear thermodynamics [10] pertain to the range of far-from-equilibrium macroscopic physics, from small- to large-scale dissipative processes and structures.

Thermodynamics regards all driven systems as mechanisms for channeling and dissipating energy [11,12], without distinguishing between animate and inanimate systems. It is not unthinkable to associate thermodynamic evolution with energy-dissipative processes. Simon Black astutely observed in 1978 [13] that life is one of many natural processes performing a dissipative role. He suggested that accelerating the dissipation of free energies accounts for the immense catalytic power of enzymes. Still, a debate continues regarding the connection between the thermodynamic aspects of biological evolution and the acceptance of thermodynamic evolution (often referred to as physical, cosmic, or universal evolution) in life sciences.

The “evolution-coupling hypothesis” was mentioned in papers and one book published by the author from 2003 to 2021 [14,15,16,17,18]. It refers to the proposal that the origin of enzymes’ prodigious catalytic power [19] is a synergy between thermodynamic and biological evolution. The biological evolution of enzymes is firmly established, but that is not the case with the thermodynamic evolution of enzymes or postulated synergy. Distinguished researchers in the field of enzyme evolution are not familiar with the concept of thermodynamic evolution. This concept is well known to researchers exploring crystal growth, transport processes in chemically reacting flow systems, plasma physics, geology, planetary sciences and climatology, the evolution of nanoparticles, self-assembly evolution, and star and galaxy formation astrophysics. For instance, the entropy production rate per unit area governs pattern formation during crystallization [20], while the evolution behavior of entropy production is relevant for controlling the work of heat engines [21]. The thermodynamic evolution is often examined during molecular dynamics simulations. One such example is the MD simulation of metamorphic protein [22].

Evolution, as a physical concept, is an energy transfer process subject to the principle of least action [23]. Robert Endres [24] used the stochastic least action principle to show how it can predict a system’s evolution in terms of thermodynamics. Without external driving forces, the thermodynamic evolution, as an energy transfer process, leads toward an equilibrium [25]. The surrounding forces drive the evolution by diminishing potential energy gradients and causing dissipative flows. Physical evolution is the response to the thermodynamic imperative of dissipating gradients. In the presence of strong enough driving forces, the spontaneous emergence of ordered structures results in more entropy production [26]. The thermodynamic concept of dissipative structures’ evolution far from equilibrium involves internal nonlinear dissipative processes and the matter and energy fluxes into and out of an open subsystem [27]. Life is a far-from-equilibrium thermodynamic phenomenon that is adept at selecting and establishing quasi-stable dissipation patterns when order and entropy advance together [28]. We can conclude the previous paragraphs by clarifying that thermodynamic evolution is the physical foundation of the evolutionary theory regardless of whether the system is abiotic or contains biotic components [11]. However, we do not question the laws of biological evolution insofar as these laws agree with the laws of thermodynamics. Our goal is to explore the contribution of thermodynamic forces and corresponding dissipative fluxes to the performance gains of enzymes that occurred during their biological evolution.

### 1.2. On the Joint Evolution of Enzymes, Dissipative Fluxes, and Catalytic Performance Parameters

We utilize the traditional approach to free-energy dissipation in enzymatic reactions [7]. Entropy production per unit of time is labeled as P and referred to as entropy production. The dissipation function is denoted as ϕ and referred to as dissipation, even when expressed in RT units. When there is only a single driving force, the product of the force X and flux J defines the dissipation function as ϕ = X∙J. Under isothermal conditions (molar-scale macroscopic description), dissipation equals the absolute temperature T multiplied by the entropy production P. Identifying partial dissipation with the bilinear expression *J_k_X*_k_ for the elementary process *k* is universally accepted in chemistry and biochemistry. The summation of such bilinear forms to calculate overall entropy production is essential in connecting microscopic and macroscopic thermodynamics [29]. We can view the total entropy production of the system at any given time as a measure of the rate of thermodynamic evolution and the system’s distance from thermodynamic equilibrium.

In the context of enzymes in living cells, entropy produced during catalysis is exported to the environment. Under homeostatic conditions, there is an almost perfect balance between the power harvested by the cell and the exported dissipation. Thus, short-term periods of homeostasis can be approximated with the steady-state condition when the thermodynamic evolution of the environment is faster due to the presence of life [30] and dwarfs the speed of biological evolution. During catalysis, enzyme conformation changes cyclically, but there is no permanent change in the structure. Also, changes in the concentrations of substrates and products are quickly rectified to maintain the cell’s preferred levels. We can compare the intensity of free-energy transduction among mitochondria, chloroplasts, bacteria, and an equivalent volume of a Sun-like star. The energy rate density is many orders of magnitude higher for living systems [31,32,33,34]. For more extended periods (eons), living systems evolve much faster than mature stars. Thus, we can ask whether there is an explicit connection between their evolution and the increase in dissipation in their environment.

We can simplify and focus the question by considering what biological evolution has achieved in the enzyme’s performance parameters and in perfecting their dissipation abilities. From a biochemical perspective, the metabolism distinguishes living cells from viruses and complex inanimate systems. The metabolism regulates the interaction with the environment, the selection of what enters and exits the cell, and almost all internal processes. Without enzymes, there would be no metabolism. Our understanding of how enzymes function has made impressive advances, but the question of how enzymes enhance the speed of chemical reactions by many orders of magnitude is still the subject of lively discussion [35,36,37]. During past decades, kinetic and free-energy descriptions of how enzymes work have been supplemented with dynamics [38,39] and stochastic thermodynamics [40,41], while the role of dissipation (if any) remained controversial [42,43]. The search for universal kinetics–dissipation relationships and the evolution of enzymes within the context of increased dissipation and catalytic specificity has remained mainly outside the focus of mainstream research. However, many essential biochemical reactions occur far from thermodynamic equilibrium, implying high dissipation [44]. Heat and entropy increase in the environment as the universal byproduct of all biological phenomena [45].

Almost 50 years ago, Roy Jensen postulated that the evolution of enzymes with high catalytic power and specificity arose from primitive enzymes possessing broad specificity and low activity [46]. The dissipation of enzymes is also subject to evolution. For one example of evolution-related enzymes, we found an intriguing, almost linear increase in overall dissipation for more evolved enzymes (closer to perfect specialized enzymes) with a higher evolutionary distance to a putative common ancestor. We also examined the laboratory evolution example [47] and found that a specialist mutant enzyme exhibited double the total dissipation and higher k_cat_ compared to its generalist relative. Thus, the extension of Jensen’s proposal [46] is possible within the framework of dissipation. The dynamical aspects of enzymes’ specificity developed through various dissipative fluxes. We discussed in previous publications [16,19,48,49] how rate-limiting steps during catalytic cycling and enzyme–substrate or enzyme–product interactions depend on the physical movements of electrons, protons, atoms, and peptide loops.

The simplest kinetic models describe cyclic reactions with one, two, or three intermediate conformational states between the substrate interacting with the free enzyme and the product release step connected to the recovery of the free enzyme. Frequently measured kinetic parameters include the turnover number k_cat_ and catalytic efficiency k_cat_/K_m_ [50]. The implicit assumption is that energy dissipation is not the primary concern. Overall dissipation can be measured as released heat [51,52,53] or calculated if all microscopic rate constants are known in the forward and reverse directions [7]. The steady-state assumption is essential to simplify the calculations of catalytic performance parameters and dissipation [19]. The rarity of such measurements and calculations impedes finding regular relationships between kinetic performance parameters and corresponding entropy production.

Enzymes performing substrate-to-product conversion can always be associated with an overall entropy production, no matter how far they work from the thermodynamic equilibrium [7]. There have been studies of dissipation for individual enzymes when chosen enzymes or molecular nanomotors were of particular interest [19,54,55,56]. Also, there have been dissipation studies for essential biochemical pathways in microbial metabolism [43,57], but we could not find anything in between. Thus, we sought to collect a database containing experimentally determined or estimated complete sets of microscopic rate constants for the simplest kinetic models. The database included 51 enzymes associated with 58 reactions for the case of the reversible cyclic Michaelis–Menten mechanism (see Supplementary Materials). We assumed a quasi-steady chemiostatic state and calculated all kinetic and thermodynamic parameters of interest, including overall dissipation ϕ and performance parameters such as the turnover number and catalytic efficiency.

Next, we examined the relationships between the enzyme’s performance parameters (kinetics) and associated dissipation. The catalytic constant k_cat_ and net flux J can differ by an order of magnitude. Thus, for fixed force X in the XJ expression, it is still far from given that the dissipation must be proportional to k_cat_. There is no physical reason to expect that the net flux J or overall entropy production will be some functions of k_cat_ or k_cat_/K_m_. However, we shall show the proportionality between dissipation and the catalytic constants of enzymes from our database. The unexpected finding is the power-law proportionality in the log-log representation of ϕ versus catalytic efficiency k_cat_/K_m_. Why would catalytic efficiency ever be proportional to dissipation when all physical definitions of thermodynamic efficiency lead to the conclusion of inverse proportionality to dissipation? Our recent publication [19] explained this paradox. The paradox does not exist, because the biochemical definition of catalytic efficiency is not equal to the definition of thermodynamic efficiency. The biological evolution resulted in an extensive range of k_cat_ and k_cat_/K_m_ values [58].

We also used synthetic data after introducing stochastic noise. Examining the relationship between dissipation and catalytic specificity for each enzyme-catalyzed reaction is possible with the artificial data. The manner of introducing stochastic noise influences the results [19]. For this work, we first chose to show that almost perfect proportionality exists between dissipation and k_cat_/K_m_ for all 58 reactions for the cases when, in each simulation step, the stochastic noise was different but identical for each forward microscopic rate constant. Secondly, our work demonstrates that the maximum entropy production requirement led to higher optimal k_cat_ or k_cat_/K_m_ from the observed performance parameters when we introduced stochastic noise only in the enzyme–substrate association and enzyme–product dissociation rate while the total force remained fixed.

Overall, our results have several repercussions. The first opens a new avenue for exploring the molecular origin of scaling laws [59]. We shall mention in the Discussion how serious the remaining challenges are in proceeding with research toward that goal. Our work’s second and most important outcome is demonstrating how universal (thermodynamic) and particular biological evolution helped each other. It is hardly a coincidence that the most evolved enzymes [60,61] (named “perfect” or highly specialized enzymes) are associated with the highest dissipation. The performance gains of enzymes during biological evolution correlate with their dissipation due to a system’s evolution in terms of thermodynamics. This result supports the interrelationship between physical (thermodynamic) and biological evolution, which we call the evolution-coupling hypothesis.

## 2. Materials and Methods

### 2.1. Statistical Analysis and Software Tools

We used Excel and Python (https://www.python.org/) tools for regression analysis. We obtained the same results for the best-fit function, the determination coefficient, and the confidence interval for the exponent of the best-fit power function by utilizing corresponding tools in Excel and Python. For sensitivity analysis, we used the Python bootstrap re-sampling tool. The enzyme performance parameters and dissipation results from our dataset (see Dataset S1 in the Supplementary Materials) are not normally distributed. Thus, we used a non-parametric Mann–Whitney U test from Python to judge whether the association of specialized enzymes with higher dissipation was statistically significant. We employed the Excel and Paint tools for the construction of figures. We created FORTRAN programs to simulate the transitions among steady states for all 58 enzyme-catalyzed reactions from our dataset in cases of (a) standard state with all parameters derived or estimated from experiments (see Dataset S1 in the Supplementary Materials), (b) forward variations simulation (see below), and (c) trade-off simulation (see below).

### 2.2. Equations for k_cat_, k_cat_/K_m_, and the Dissipation Function

**Figure 1:**
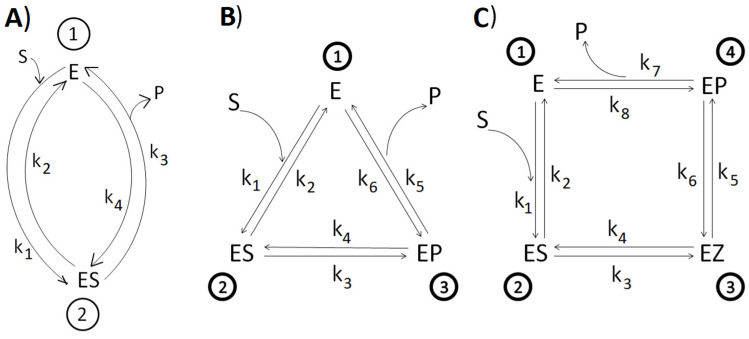
Reversible kinetic schemes for transitions among functionally important enzyme conformations. We assumed predominantly counterclockwise cycling among states E (1), ES (2), EP or EZ (3), and EP (3 or 4). Forward kinetic constants are k_1_, k_3_, k_5_, and k_7_. The reverse kinetic constants are k_2_, k_4_, k_6_, and k_8_. (**A**) Two-state scheme. (**B**) Three-state scheme. (**C**) Four-state scheme. We multiplied the second-order rate constants by the substrate or product concentration to obtain k_1_ and k_4_ in panel (**A**), k_1_ and k_6_ in panel (**B**), and k_1_ and k_8_ in panel (**C**). In this way, all rate constants are expressed in the units of inverse seconds (first-order rate constants).

We used well-known equations [62,63] for forward (S → P) Michaelis constants and maximal activities in the case of two-, three-, and four-state cyclic kinetic mechanisms (Figure 1). Substrate and product concentrations are mostly not explicitly present in the following equations because they were multiplied by the second-order rate constants. For the two-state reversible cycle (Figure 1A)

(1) $$k_{cat}=k_{3}\;\mathrm{and}\;\frac{k_{cat}}{K_{m}}=\frac{k_{1}k_{3}}{\left[S\right](k_{2}+k_{3})}$$

For the three-state cyclic kinetic mechanism (Figure 1B)

(2) $$k_{cat}=\frac{k_{5}}{1+\frac{k_{4}}{k_{3}}+\frac{k_{5}}{k_{3}}}\;\mathrm{and}\;\frac{k_{cat}}{K_{m}}=\frac{k_{1}k_{3}k_{5}}{\left[S\right](k_{2}k_{4}+k_{2}k_{5}+k_{3}k_{5})}$$

For the four-state cyclic kinetic mechanism [64] (Figure 1C)

(3) $$k_{cat}=\frac{k_{3}}{1+\frac{k_{3}}{k_{7}}+\frac{k_{3}}{k_{5}}\left(1+\frac{1}{K_{2}}\right)\left(1+\frac{1}{K_{3}}\frac{k_{5}}{k_{7}}\right)}$$

and

(4) $$\frac{k_{cat}}{K_{m}}=\frac{{k_{1}k_{3}k_{5}k}_{7}}{\left[S\right](k_{2}k_{4}k_{6}+k_{2}k_{4}k_{7}+k_{2}k_{5}k_{7}+k_{3}k_{5}k_{7})}$$

All of these equations are strictly valid only for uni-uni enzymatic reactions. However, Karamitros et al. [47] used the same four-state parameters, k_cat_ and K_m_ (Equations (3) and (4)), when one substrate enters the reaction and two products exit it. These authors utilized the symbol k_1_ for the second-order substrate-binding constant, which is k_1_/[S] in our notation (see Section 3.1). Thus, we also used those equations for the four-state catalysis mechanism of carbonic anhydrases, soluble inorganic pyrophosphatase, and kynureninases when two products enter or exit the reaction. We often used the maximal reaction rates and the Michaelis constant in the forward and reverse directions for two-state reversible kinetic models to find all four microscopic rate constants (see Section 2.4).

With all of the microscopic rate constants known, we followed Terrell L. Hill [7] in finding the expressions for the single-cycle forces and fluxes in a steady state. The two-state expressions for net reaction flux J and the thermodynamic force X are

(5) $$J=\frac{k_{1}k_{3}-k_{2}k_{4}}{k_{1}+k_{2}{+k}_{3}+k_{4}}\;\mathrm{and}\;X=RTln\frac{k_{1}k_{3}}{k_{2}k_{4}}=RTlnK$$

respectively, where K = K_1_∙K_2_ is the equilibrium constant. The three-state expressions are

(6) $$J=\frac{k_{1}k_{3}k_{5}-k_{2}k_{4}k_{6}}{k_{1}\left({k_{3}+k_{4}+k}_{5}\right)+k_{2}k_{4}+k_{2}k_{5}{+k}_{3}k_{5}+k_{6}\left({k_{2}+k_{3}+k}_{4}\right)}$$

and

(7) $$X=RTln\frac{k_{1}k_{3}k_{5}}{k_{2}k_{4}k_{6}}=RTlnK$$

with K = K_1_∙K_2_∙K_3_.

The four-state expressions are

(8) $$J=\frac{k_{1}k_{3}k_{5}k_{7}-k_{2}k_{4}k_{6}k_{8}}{\Sigma_{1}+\Sigma_{2}+\Sigma_{3}+\Sigma_{4}}$$

with

$$\Sigma_{1}=k_{2}k_{4}k_{6}+k_{2}k_{4}k_{7}+k_{2}k_{5}k_{7}+k_{3}k_{5}k_{7}\;\Sigma_{2}=k_{1}k_{5}k_{7}+k_{4}k_{6}k_{8}+k_{1}k_{4}k_{6}+k_{1}k_{4}k_{7}\;\Sigma_{3}=k_{1}k_{3}k_{7}+k_{2}k_{6}k_{8}+k_{3}k_{6}k_{8}+k_{1}k_{3}k_{6}\;\Sigma_{4}=k_{2}k_{4}k_{8}+k_{1}k_{3}k_{5}+k_{3}k_{5}k_{7}+k_{2}k_{5}k_{7}$$

and

(9) $$X=RTln\frac{k_{1}k_{3}k_{5}k_{7}}{k_{2}k_{4}k_{6}k_{8}}=RTlnK$$

where K = K_1_∙K_2_∙K_3_∙K_4_.

The dissipation function ϕ is then the J∙X product in each case. We used the convention of treating all microscopic rate constants equally as first-order constants (see Section 3.1). The identity and concentration of the substrate and product or products can be found in the Dataset S1 Excel file and source codes from the Supplementary Materials.

### 2.3. Introducing Normal Noise in Microscopic Rate Constants

In forward variations, we multiplied each of the observed or estimated forward rate constants k_i_ with the Box–Muller transform [65]:

(10) $$g_{i}=\sqrt{-2lns_{1}}\cos\left(2\pi s_{2}\right)+1.0$$

where *s*_1_ and *s*_2_ are random numbers chosen from the unit interval (0, 1) by the standard FORTRAN generator *random_number*. The shift +1.0 gives prominence to positive numbers for modified rate constants. Since rate constants k_i_ > 0, we used only positive g_i_ values for the simulations. The simulations had 1000 to 30,000 steps, resulting in the same number of rows in the program’s output. Rows with negative random numbers were replaced with the first previous row with a positive random number. In all reactions that we examined, there was an almost perfect linear increase (the determination coefficient close to 1.0) in the enzyme efficiency k_cat_/K_m_ (vertical axis) as a function of the dissipation/(RT) variable (horizontal x-axis). The state probabilities, information entropy, Michaelis constant, equilibrium constants, and force did not change during the simulations that we named forward variations. The constancy of the equilibrium constants K_i_ was imposed from the outset. The slope of the best-fit line for the efficiency versus dissipation function decreased from close to 90 degrees to approximately 45 degrees as the overall dissipation decreased for 58 reactions from our dataset (see Results chapter and Dataset S1 in the Supplementary Materials).

In trade-off variations, we allowed for the compensatory changes in the equilibrium constants for the enzyme–substrate association and the last reaction step of the enzyme–product dissociation. We also kept the total force constant X in these simulations. We did not derive the general proof that the maximum in the entropy production can always be found, but we did find that maximum in all 58 reactions from our dataset (see Results and Dataset S1). We had to increase the substrate concentration to reach the maximum in four reactions (see Results). Once the steady state with maximum dissipation is found, all other (optimal) parameters can be compared with their estimated or observed values. Either the catalytic constant or enzyme efficiency is higher for the maximal dissipation state. The maximal k_cat_/K_m_ for trade-off variations is associated with similar or smaller dissipation from the observed value (see Results for one example). We deposited the programs required to build the figures on GitHub (https://github.com/DJureticSplit/PERF-ENZYMES) (accessed on 27 March 2025). See the Supplementary Materials for examples of source codes performing calculations of all parameters using the experimental results and simulation types that we named forward and trade-off variations. These source codes are freely available for download.

### 2.4. The Dataset Collection

The complete set of microscopic kinetic constants in the forward and reverse directions has occasionally been measured or estimated for some enzymes with simple uni-uni kinetic mechanisms. To the best of our knowledge, there has never been a systematic effort to collect a complete set of k_i_ values for as many enzymes as possible. Moreover, the interest in publishing all k_i_ values for studied enzymes has waned over the decades, possibly because the k_cat_ and K_m_ kinetic constants (macroscopic kinetics data) were considered sufficient for the kinetic analysis. Free-energy profile determination requires the knowledge of all k_i_ values. However, free-energy profile construction remains challenging. It involves combining complex experimental techniques with sophisticated computational optimizations [64].

To collect our database, Dataset S1 (see the Supplementary Materials), we first used many different keywords and phrases in searches of Google Scholar or PubMed, combined with the word “enzyme”. Examples include “characterization”, “simulation”, “catalytic properties”, “catalytic rate”, “rate constants”, “microscopic rate constants”, “energy profile”, “kinetic analysis”, “kinetics of”, “catalytic properties”, “catalytic steps”, “catalytic cycle”, “kinetic and thermodynamic”, “evolution of”, and others. After searching through hundreds or thousands of papers, none of the keywords or phrases helped find more than two to four of the articles cited in Dataset S1 [16,19,47,54,64,66,67,68,69,70,71,72,73,74,75,76,77,78,79,80,81,82,83,84,85,86,87,88,89,90,91]. Searching for some enzyme classes, such as mutases, epimerases, isomerases, and racemases, could improve the search strategy due to the simplicity of their catalytic mechanisms and the low total number of k_i_ constants (see Figure 1). The focus on two-state reaction cycles (Figure 1A) also helped obtain the desired results, due to the possibility of extracting the complete set of four k_i_ constants from the V_m_ and K_m_ parameters determined in both directions [73].

A good strategy was to examine publications by experts who appreciated the insights that complete kinetic characterization offers into enzymes’ evolution and the nature of their catalytic mechanism. To mention just a few of them, W. John Albery and Jeremy R. Knowles [60], Kenneth A. Johnson [50], and Michel D. Toney [64] took up the challenge of extracting information about enzymatic free-energy profiles from the determination of microscopic rate constants. In one search example, we used a combination of keywords, phrases, and enzyme class names for the Google Scholar search (“enzyme”, “computer simulations”, “kinetic parameters”, and “isomerase”) to find 378 hits within the default setting. A promising feature was that our recent paper [19] was the first hit because it contains a complete set of microscopic rate constants for 10 enzymes. McIntyre et al.’s paper [69] was the 19th hit, and we selected nine enzymes with determined rate constants from that publication in our Dataset S1. The next best example was finding four enzyme variants with determined k_i_ values. We used the phrase “cyclophilin rate constants” in the PubMed default search to find 63 hits. The 12th hit was Holliday et al.’s paper [78], which estimated all forward and reverse k_i_ values for the cyclophilins CypA, CypB, CypC, and GeoCyp. We may have missed some papers containing the required data in these and related searches. The BRENDA enzyme database [92] (Braunschweig enzyme database) is a rich source for enzymatic reactions. There is no obvious way to search in BRENDA for all instances when a complete set of microscopic rate constants has been determined or estimated. None of the items from Dataset S1 was first found in BRENDA or other databases. No more than 30 research papers served as the origin for the parameters that we collected in the Dataset S1 Excel table.

We did not intend to collect the required data exclusively for soluble proteins. Our database has one example of the enzyme “walking” in one dimension (kinesin-1) and several examples of immobilized enzymes. We considered them equivalent to soluble proteins for our analysis. Therefore, we also used measured microscopic rate constants for our calculations for such cases. Membrane proteins likely succumb to the same thermodynamic drive as soluble proteins. Increased complexity in calculating the performance parameters and overall dissipation for membrane enzymes influenced our choice to consider a similar analysis beyond the present manuscript’s scope.

### 2.5. The Phylogenetic Tree Construction

We collected 36 mature β-lactamase sequences in the MEGA7 tool for molecular evolutionary genetics analysis [93]. The aim was to reconstruct the evolutionary relationships among mature sequences (without signal peptides) of *Staphylococcus aureus* PC1, *Escherichia coli* RTEM, *Bacillus cereus* β-lactamase 1, and the remaining 33 β-lactamases. The UniProt database did not yet exist when Richard P. Ambler published the sequences for three β-lactamases [94]: PC1, RTEM, and Lac1. We identified the UNIPROT sequences P00807 and P62593 for the primary structures of PC1 and RTEM, respectively. Gaps and errors in Ambler’s original sequence for mature Lac1 were corrected [95] before phylogenetic tree construction. The maximum likelihood method in MEGA7 was based on the JTT matrix-based model [96]. Initial trees for the heuristic search were obtained automatically by applying the neighbor-joining and BioNJ algorithms to a matrix of pairwise distances estimated using a JTT model. We then selected the topology with a superior log-likelihood value. There were a total of 169 positions in the final dataset after the elimination of gaps and missing data. Henriette Christensen and coauthors referred to three lactamases that we highlighted with the red title “Ambler sequence” (see Results) in their seminal 1990 paper about the determination of rate constants in the Michaelis–Menten 3-state kinetic scheme [97]. We calculated backward rate constants in the second and third catalytic steps by assuming that they were very low compared to the corresponding forward rate constants [16]. These preparatory steps enabled the exploration of whether an increased dissipation and evolutionary distance from a putative common ancestor of all β-lactamases accompany evolutionary gains in performance parameters.

## 3. Results

### 3.1. The Database

There are many different datasets for the performance parameters of enzymes, but we are not aware of any dedicated to enzymes with all forward and reverse microscopic rate constants known. We used different search strategies to collect as many enzyme-catalyzed reactions from published papers containing such data as possible (see Section 2.4). However, the need for the complete set of observed or estimated microscopic rate constants severely restricted the outcome. We additionally limited the search to cyclic enzyme reactions without branching, involving two, three, or four conformational states (Figure 1).

**Figure 2:**
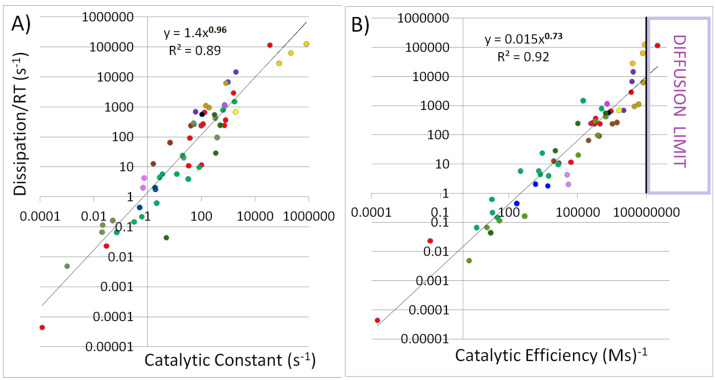
Multi-colored results for the main enzyme classes (EC numbers). Enzyme names are available in Table 1. Corresponding references are available in Dataset S1. (**A**) We illustrate a roughly linear relationship between entropy production (dissipation) and catalytic constant (k_cat_) for 58 enzyme-catalyzed reactions from our database. (**B**) The right-hand panel exhibits the power-law relationship with the 0.73 exponent between the dissipation and enzyme efficiency (k_cat_/K_m_) for the same reaction set. The determination coefficient values of 0.89 (**A**) and 0.92 (**B**) indicate a good-to-solid fit in a linear regression model for the log-transformed data. The 95% confidence interval (CI) for the exponent is (0.87, 1.05) for (**A**) and (0.67, 0.78) for (**B**). These results mean that the exponent is reliably close to 0.96 (**A**) and 0.73 (**B**), with high confidence that the relationship between enzyme performance and dissipation follows a power law with an exponent in this range. The sensitivity analysis using the bootstrap method yielded a mean exponent of 0.731, a standard deviation of 0.033, and a 95% CI range of 0.67–0.80 for the (**B**) regression line. The diffusion limit range (pink rectangle) from (**B**) starts from 10^8^ (Ms)^−1^. It highlights several specialized enzymes (see the main text) that reached the pinnacle of their evolutionary development for their catalytic efficiency and associated high entropy production. Seven generalist enzymes are among those catalyzing the 20 reactions with the lowest dissipation (less than 6 s^−1^ dissipation/RT), and only one is among the enzymes catalyzing the 20 reactions with the highest dissipation. The chosen colors are red for isomerases (EC 5.2.1.8, 5.3.1.-, and 5.3.3.1), green for racemases (EC 5.1.1.-, and 5.1.2.2), dark olive green for epimerases (EC 5.1.1.21, and 5.1.3.-), olive for mutases (EC 5.4.2.1, 5.4.3.6, and 5.4.99.-), pink for kynureninases (EC 3.7.1.3), psychedelic purple for β-galactosidases (EC 3.2.1.23), purple for β-lactamases (EC 3.5.2.6), yellow for fumarate hydratases (EC 4.2.1.2), orange for carbonic anhydrases (EC 4.2.1.1), bronze for soluble inorganic pyrophosphatases (EC 3.6.1.1), blue for (R)-selective amine transaminases (EC 2.6.1.21), and black for kinesin-1 (EC 5.6.1.3).

Nevertheless, the presented database (see Table 1 and Figure 2 in the main text and Dataset S1 in the Supplementary Materials) contains 58 reactions catalyzed by 51 enzymes and 9 mutated enzymes, which belong to four out of the seven main enzyme classes in the IUBMB enzyme classification system for EC numbers. There are 13 enzymes named isomerases, 12 racemases, 8 mutases, and 4 epimerases, all belonging to the EC 5 family of isomerases. Kinesin-1 (black point in Figure 2) is also classified as an isomerase (EC 5.6.1.3) because its motor function is based on the cyclical changes in protein conformation. There are 30 reactions involving only two conformational states of enzymes, and 14 reaction mechanisms with three states (Figure 1A,B). The remaining 14 reactions go through four states: free enzyme E, enzyme–substrate complex ES, intermediate complex EZ, and enzyme–product complex EP (Figure 1C).

Reported microscopic rate constants were observed or estimated in experiments with isolated enzymes by choosing the optimal conditions for each enzyme. Accordingly, it was impossible to avoid different conditions for each enzyme, such as temperature, pH, and substrate-to-product ratio. All transitions between enzyme conformational states were treated as first-order processes. For example, for the binding transition E + S → ES, we used $k_{1}=k_{1}^{*}[S]$, where $k_{1}$ is the first-order rate constant (in s^−1^ units), $k_{1}^{*}$ is the second-order rate constant (in M^−1^s^−1^ units), and [S] is the concentration of the substrate (in moles). The same notation was used for product-binding transitions. This is why the substrate concentration is present in k_cat_/K_m_ expressions (Equations (1), (2) and (4)). Substrate or product concentrations were multiplied by the second-order rate constants to obtain, for instance, four forward rate constants (k_1_, k_3_, k_5_, and k_7_) and four backward rate constants (k_2_, k_4_, k_6_, and k_8_) in the s^−1^ units (Figure 1C). The associated equilibrium rate constants K_1_ = k_1_/k_2_, …, K_4_ = k_7_/k_8_ represent a non-equilibrium situation for the out-of-equilibrium system. The simple scheme in Figure 1C has several exceptions when two products exit the cycle (see Section 2.2). We calculated the performance parameters k_cat_ and K_m_ using equations for forward reactions (see Section 2.2). The calculated kinetic parameters were similar to the published values in the papers where we found the microscopic rate constants. Our choice of product concentration ensured the forward direction for each reaction. The driving force X and corresponding net flux J were then both positive. That choice resulted in a range of X/(RT) values from 1 to 23. The range of J values extended from a minimal 10^−4^ (for glucose isomerase) to a maximal 4 × 10^5^ s^−1^ (for carbonic anhydrase II). The total dissipation was calculated [7] and expressed in inverse seconds, as described in the Section 2.2.

We assumed a non-equilibrium steady state for each of the 58 reactions with constant enzyme, substrate, and product concentrations and a fixed driving force X (Table 1 and Dataset S1). Examples of highly driven far-from-equilibrium reactions with X/RT greater than 10 are for the ALiO, PC1, KIN, and AROH enzymes (abbreviations are defined in Table 1). The single-cycle enzymes from the database cannot perform free-energy transduction [7]. They are not interconnected in a metabolic network, because we selected them from different life domains (mostly from bacteria). Still, each enzyme opens a specific channel for the dissipative production of essential metabolites. Single-cycle enzymes are crucial facilitators and regulators of metabolic energy flow, even though they ultimately dissipate all free energy as heat. The database contains several exceptions to soluble enzymes. Kinetic data that we used for glucose isomerase and β-galactosidase are for immobilized enzymes [40,90,91]. Kinesin-1 mainly performs one-dimensional motion along microtubules [75].

### 3.2. The Regular Relationships Between Dissipation and the Performance Parameters of Enzymes

To test for the presence of power-law scaling

(11) $$\frac{Dissipation}{RT}=\alpha {\left\{\frac{k_{cat}}{K_{m}}\right\}}^{\beta }$$

we started with the null hypothesis that no significant relationship exists between $\log_{10}\frac{k_{cat}}{K_{m}}$ and $\log_{10}\frac{Dissipation}{RT}$. We next performed the linear regression on log-transformed data for columns A (x-values k_cat_/K_m_) and C (y-values dissipation/RT) from Dataset S1 (corresponding to columns highlighted with a gray background in Table 1). The regression results were as follows: intercept log(α) = −1.82, slope (β) = 0.728, R^2^ (goodness of fit determination coefficient) = 0.922. The *p*-value for the slope was 0.000, far below the 0.05 threshold, allowing us to reject the null hypothesis. The relationship between $\log_{10}\frac{k_{cat}}{K_{m}}$ and $\log_{10}\frac{Dissipation}{RT}$ was statistically significant. In other words, the data from Dataset S1 (columns A and C) exhibit a statistically significant power-law scaling with a high degree of fit. 

We performed the same statistical analysis for the log-transformed data from columns B and C (corresponding to the k_cat_ and ϕ/(RT) columns in Table 1). The regression analysis results for the relationship between $\log_{10}k_{cat}$ (x-variable) and $\log_{10}\frac{Dissipation}{RT}$ (y-variable) were as follows: intercept log(α) = 0.149, slope (β) = 0.962, and R^2^ = 0.893. The hypothesis of no significant linear relationship between variables x and y (null model) was rejected, with the *p*-value = 0.000 for the slope. The data exhibit a power-law scaling of approximately $y=x^{0.962},$ with a very good fit.

Figure 2 illustrates the power-law proportionality between dissipation and performance parameters for 58 reactions catalyzed by soluble enzymes. The best-fit lines look similar in the log-log graphs from both panels in Figure 2. However, for most reactions, the enzyme efficiency values k_cat_/K_m_ are several orders of magnitude higher than the dissipation values. In contrast, the dissipation and the catalytic constant values (k_cat_ at the x-axis) are similar. This is due to the high specificity of enzyme–substrate interactions, which leads to small values of the Michaelis–Menten constant K_m_. The dissipation values in Figure 2 range from extremely small to exceptionally high for enzymes that are explicitly named isomerases (red points), racemases (green points), mutases (olive points), and epimerases (dark olive green points).

At the lower end of the spectrum are reactions catalyzed by enzymes producing less than 10 s^−1^ dissipation/(RT). The K_m_ values that we found for such enzymes (Dataset S1) indicate that most are not highly specific for their substrate. For instance, the generalist and versatile enzymes exhibiting low dissipation in Figure 2 are proline racemases from archaea (EC 5.1.1.4, green points) [83], the HsKYNase_93D9 variant of human kynureninase (EC 3.7.1.3, pink point) [47], TM0831 racemase (EC 5.1.1.-, green point) [86], and glucose isomerases (EC 5.3.1.5, red points) [90,91].

The points for the most evolved specialist (perfect) enzymes are close to or inside the diffusion-limited region of Figure 2B when k_cat_/K_m_ is similar to or ≥10^8^ (Ms)^−1^ (the right-hand highlighted region of Figure 2B). Such reactions and associated enzymes exhibit high dissipation/(RT) values between 6000 and 125000 s^−1^. From our database, carbonic anhydrase II from human red blood cells (EC 4.2.1.1, orange points), ketosteroid isomerase from *Commamonas testosteroni* (EC 5.3.3.1, red point), and soluble inorganic pyrophosphatase from *Streptococcus gordonii* (EC 3.6.1.1, chocolate point) are specialized enzymes with the highest enzyme efficiency and dissipation. Within our limited data (Dataset S1), no clear preference exists for extremes in dissipation or enzyme efficiency in any of life’s kingdoms. Still, we found low dissipation for the bifunctional proline racemases derived from an ancestral archaeal enzyme [83].

Our dissipation and enzyme performance parameters data (Table 1 and Dataset S1) are not normally distributed. We applied the non-parametric test to determine the significance of the distribution of dissipation numbers among specialized and generalist enzymes. The null hypothesis was that the numbers (dissipation values) are distributed randomly between two enzyme classes. However, the *p*-value of 0.008 supports the non-randomness of the data allocation and suggests a statistically significant difference. Thus, there is strong statistical evidence that the category of specialized enzymes is associated with higher entropy production (see Section 2.1).

The linear regression model for the log-transformed data demonstrated a good fit for the power functions y = 1.4∙x^0.96^ (Figure 2A, R^2^ = 0.89) and y = 0.015∙x^0.73^ (Figure 2B, R^2^ = 0.92). The calculated confidence intervals for the exponent are relatively narrow (see the legend in Figure 2). The sensitivity analysis (re-sampling the data multiple times and re-running the regression) showed minimal sensitivity. It confirmed the robustness of the exponent estimate at 0.96 and 0.73 for dissipation’s dependence on the catalytic constant and enzyme efficiency, respectively. A 95% confidence interval (0.67–0.80) for the exponent from bootstrap analysis indicated that the power law for the dissipation–efficiency dependence with the 0.73 exponent (Figure 2B) is stable even under variations in the data.

### 3.3. Can Regular Dissipation–Performance Relationships Be Obtained for Individual Reactions After Introducing the Stochastic Noise to Obtain Synthetic Data?

We assumed that all 58 of the studied enzyme–ligand systems can jump among quasi-steady states in stochastic ways. Stochastic models are a simple way to create artificial data for individual reactions and to imitate biological mechanisms that harness molecular noise to create variable beneficial outcomes [19,98]. Stochastic fluctuations are always present in biological situations with a small number of molecules interacting in small volumes. The random noise that we introduced in microscopic rate constants does not imitate actual fluctuations in any biological situation. Instead, it is a theoretical method to examine the influence of random variations. The simplest assumption is an equal effect of variations on all forward rate constants. We refer to “forward variations” when we use that assumption.

For fixed equilibrium constants, K_i_, when overall force was also fixed, we called Gaussian noise (see Section 2.3 and FORTRAN source codes from Supplementary Material). We then multiplied each forward kinetic constant for a chosen reaction with a random number from normal noise. An almost perfect linear proportionality between dissipation and enzyme efficiency exists for all 58 reactions, and it does not depend on the choice of enzyme or the number of conformational states in the cyclic scheme. Juretić and Bonačić Lošić [19] found the mathematical reasons why an excellent proportionality should always be expected upon introducing the noise as described above. Next, we examined the maximal values in all parameters. Each parameter of interest (forward rate constants k_i_ (odd i), catalytic constant k_cat_, enzyme efficiency k_cat_/K_m_, dissipation/RT) significantly increased above the experimental value (four to five times). This theoretical result does not have any special physical meaning. All the same, catalytic optimizations do not contradict any physical law if similar or equal noise is introduced in each catalytic step.

We can also examine the assumption that the substrate–enzyme association and enzyme–product dissociation step in the presence of noise are subject to the trade-off regulation due to concerted protein dynamics. These are “trade-off variations”. We introduced the stochastic noise, but only in the enzyme–substrate and enzyme–product equilibrium constants, while total force remained fixed, and we looked for the maximum entropy production states. Several observations followed after exploring all 58 reactions from our dataset within the framework of the trade-off variations. Firstly, the maximum in the dissipation always exists. Furthermore, compared to the dissipation calculated from the observed data, the maximum always has a higher value. There is a 1% or less difference between the maximal dissipation and the dissipation calculated from the experimental data for six reactions (GI, NSAARN, API, EpiI, FH, and ssPPase). Thus, in some cases, the maximum entropy production requirement comes close to reproducing the observed kinetic and thermodynamic parameters. Still, there are three reactions with about 10 times higher dissipation and 17 to 22 times higher optimal k_cat_ (HcmABwt, GeoCyp, and ALF). Secondly, the optimal enzyme efficiency values for maximal dissipation are higher than the observed value for 24 reactions (Eff-enzyme reactions) but smaller than that for 34 reactions (Tur-enzyme reactions). The optimal k_cat_ (from the maximal dissipation requirement) is smaller for all Eff-enzyme reactions than the observed catalytic constant. The reverse holds for the Tur-enzyme reactions when optimal *k_cat_* > $k_{cat}^{exp}$. Our division of all enzymes into two classes (see Table 2 and Dataset S1) can serve as a guide when deciding whether to improve enzymes’ turnover number (Tur-enzymes) or their efficiency (Eff-enzymes). Thus, improving some enzyme performance parameters using the physical principle of maximum entropy production depends on an informed choice of appropriate reactions, the kinetic parameter (for optimization), and the selection manner for an optimal steady state.

**Figure 3:**
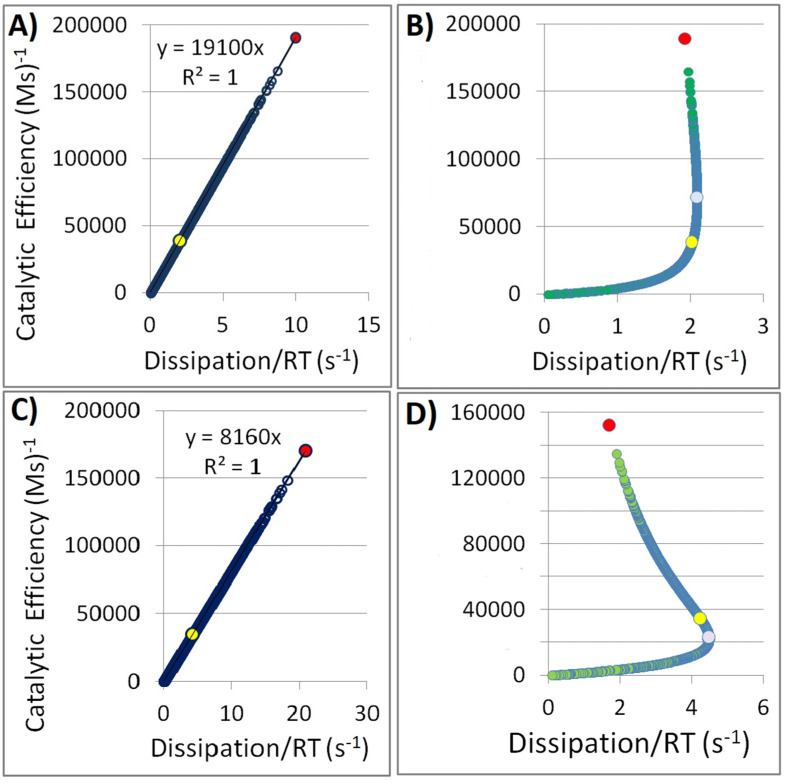
Experimentally observed and simulation results for medically important variants of human kynureninase [47]: the generalist variant HsKYNase_93D9 (**A**,**B**), and the specialized variant HsKYNase_66 (**C**,**D**). The dissipation calculated for the specialist variant from the observed data is double that of the generalist variant (compare yellow points in the upper and lower panels). The simulations using forward variations (**A**,**C**) illustrate an almost vertical linear increase in the enzyme efficiency k_cat_/K_m_ for small changes in the dissipation/RT values when all forward rate constants are subject to the same random noise within the restriction that the equilibrium constants K_i_ do not change in the simulation steps. The yellow and red points represent the observed and maximal k_cat_/K_m_ values, respectively. The trade-off variations provided different results (**B**,**D**). The maximum entropy production requirement for the trade-off between enzyme–substrate association and enzyme–product dissociation rate within the framework of fixed total force almost doubled the optimal enzyme efficiency ((**B**), light pink point) with respect to the observed value ((**B**), yellow point) for the generalist variant. For the specialized variant, trade-off variations resulted in a smaller optimal k_cat_/K_m_ value ((**D**), light pink point) with respect to the observed value ((**D**), yellow point). The yellow and red points have the same meaning and similar k_cat_/K_m_ values as in (**A**,**C**).

For example, we asked whether noise introduction can improve the performance of medically important human kynureninase. The generalist variant HsKYNase_93D9 and the specialized variant HsKYNase_66 [47] were the starting points for our simulations (two pink points near label 1 for the k_cat_ axis in Figure 2A, and near label hundred thousand 100 for the k_cat_/K_m_ axis in Figure 2B). Panels A and C in Figure 3 illustrate the significant increase in the k_cat_/K_m_ that can be gained in theory after paying the “price” of increased dissipation when the same noise is introduced in all forward rate constants. The generalist variant is the Eff-type enzyme. Thus, it exhibits a better response in the k_cat_/K_m_ values after such forward variations. Figure 3B,D illustrate a different behavior between the generalist and specialized kynureninase variants when noise introduction is restricted to the enzyme–substrate association constant k_1_ and enzyme–product dissociation constant k_7_. The fixed force restriction and trade-off variations then lead to the maximum entropy production and an optimal k_cat_/K_m_ (the pink point), which is either double the observed efficiency for the generalist mutant or smaller than the observed value for the specialized mutant. 

**Figure 4:**
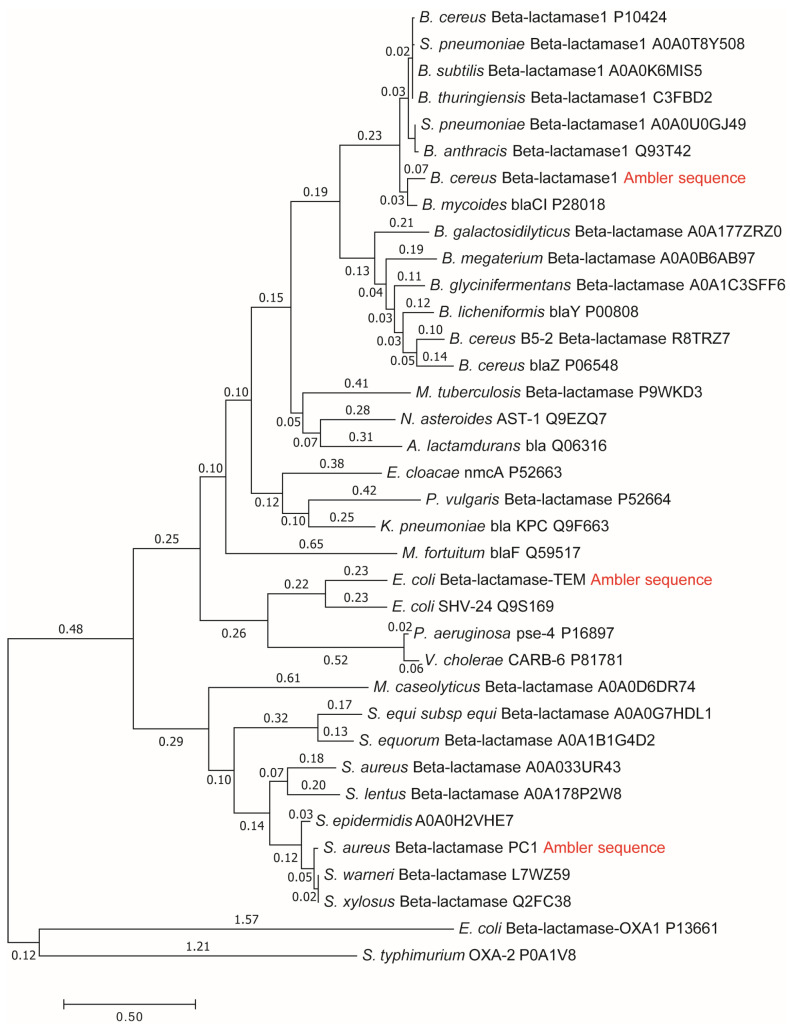
Molecular phylogenetic tree and calculation of evolutionary distances by maximum likelihood method for β-lactamases PC1, RTEM, and Lac1 [96] after using the corrected sequence of Lac1 [95]. Summing all relevant branch lengths (number above each branch leading to the label “Ambler sequence” in red color) gives the following results in evolutionary distances: 1.19 for PC1, 1.44 for RTEM, and 1.60 for Lac1 (reproduced from [17]).

**Figure 5:**
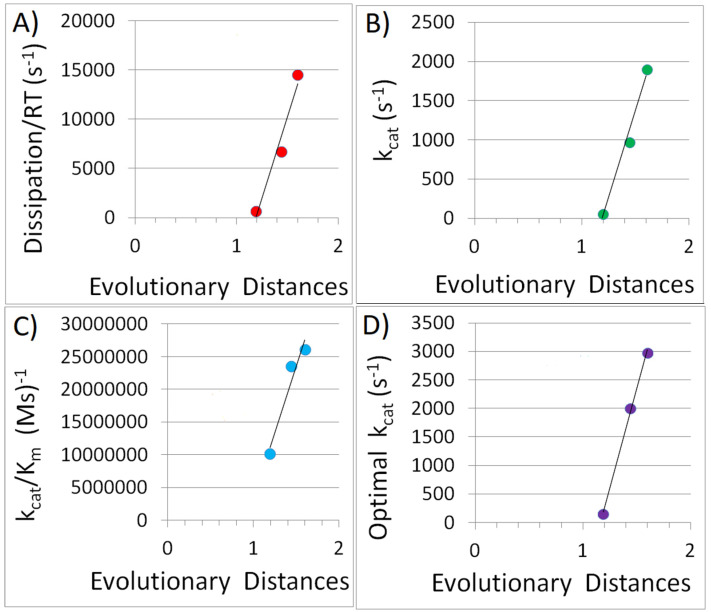
The performance parameters and evolutionary distances (from a common ancestor) of three β-lactamases: PC1 from *S. aureus*, RTEM from *E. coli*, and Lac1 from *B. cereus*. The increase in the evolutionary distance PC1 = 1.19 < RTEM = 1.44 < Lac-1 = 1.60 [16,19] is associated with the higher dissipation (**A**), catalytic constant (**B**), enzyme efficiency (**C**), and optimal k_cat_ that we derived from the maximal entropy production requirement (**D**). The PC1 (x,y) parameters define the lowest, RTEM the middle, and Lac-1 the highest point in all four panels.

Another example involves medically important enzymes known as β-lactamases. These specialized enzymes, including PC1, RTEM, and Lac-1, had their kinetic parameters established before the evolution of bacterial resistance prompted changes in their characteristics [97]. These enzymes share a common ancestor and are evolutionarily related [16,94]. We conducted a molecular phylogenetic analysis to determine the evolutionary distances from the putative common ancestor (Figure 4). We investigated whether specialized enzymes like β-lactamases exhibit higher dissipation, turnover number (k_cat_), and catalytic efficiency (k_cat_/K_m_) as their evolutionary distance from a common ancestor increases. The results shown in Figure 5A–C offer a positive answer to these questions. Since β-lactamases fall within our Tur-enzyme class, the simulated trade-off variations increased their turnover numbers, from observed rates of 61, 975, and 1905 s^−1^ to optimal values of 151, 2000, and 2977 s^−1^ for the PC1, RTEM, and Lac-1 enzymes, respectively. The relationship between optimal k_cat_ and evolutionary distance, as presented in Figure 5D, appears almost perfectly linear. However, it is worth noting that the degree of proportionality and linearity in the figures is based on only three data points. This can be viewed as a general trend and suggests that including other families of evolutionarily related enzymes in future studies would be beneficial.

## 4. Discussion

### 4.1. Is the Proportionality Between Dissipation and Kinetic Parameters of Enzymes Expected (Trivial) or a Scientifically Valuable Result?

Before interpreting Figure 2 as our central result, we should consider whether it holds scientific value. For the constant force X, one could attempt to replace the dissipation expression J∙X with the product k_cat_∙X, as the numerical values of the catalytic constant k_cat_ in the forward direction and net flux J are often similar (see Table 1). Additionally, their units are identical. However, this raises several issues.

According to the second law of thermodynamics, entropy production must be greater than or equal to zero. The net flux J can be negative when X is also negative to maintain the positivity of the J∙X product. The definition of net flux is given as follows:

(12) $$J=J_{+}-J_{-}$$

where $J_{+}$ is the forward flux and $J_{-}$ is the backward flux.

When the backward flux is greater, the net flux J becomes negative. In contrast, k_cat_ is defined as the ratio of maximum reaction velocity to the total enzyme concentration involving two quantities that are always positive, meaning that k_cat_ must be positive. A negative k_cat_ would imply that the enzyme reduces the product formation rate, which contradicts enzymes’ fundamental role as biological catalysts. Furthermore, we noted in the Introduction that k_cat_ represents the turnover rate, which is inherently a non-negative quantity.

The second issue arises when k_cat_ and J are positive but have significantly different numerical values. In forward-directed reactions, the turnover rate is always higher than the net flux J (compare the k_cat_ and J columns in Table 1, or columns B and X in Dataset S1). In several cases that we examined, the ratio k_cat_/J exceeds 10. Examples from our dataset include D-psicose 3-epimerase (ratio = 12.2), glutamate racemase mutant R25A (ratio = 18.7), glutamate racemase (ratio = 23.3), and D-psicose 3-epimerase mutant R215K (ratio = 103.4).

Finally, it is important to note that the calculation of k_cat_ does not consider the association rate constants for the enzyme + substrate and enzyme + product systems (see Section 2.2). The k_cat_ value reflects the speed of the irreversible decomposition of the enzyme–substrate complex into free enzyme and free product. The original Michaelis–Menten kinetics scheme inspired the definition of k_cat_; unfortunately, that scheme is incompatible with a thermodynamic description due to the omission of the reverse reaction E + P → ES.

Figure 2 may seem trivial if the dissipation, ϕ, is simply an analytical function of k_cat_ or k_cat_/K_m_. We should note that k_cat_ and k_cat_/K_m_, the preferred performance parameters in enzyme kinetics, have different units and meanings than entropy production, which is a fundamental physical quantity in non-equilibrium thermodynamics. Thus, it would be inappropriate to derive fundamental physics from empirical kinetic parameters that are primarily valuable for biochemistry. Moreover, Figure 2 would lack significance if other researchers had previously collected, published, and interpreted similar datasets. However, this does not appear to be the case.

We can also entertain the possibility that all of our flux and force calculations are wrong because these quantities are concentration-dependent, meaning that their values would change along the course of the reaction. With unknown concentrations, it is impossible to calculate flux, force, and entropy production for non-equilibrium conditions using Terrell Hill’s [7] or any other method. Equations (5)–(9) for flux and force calculations do not contain substrate and product concentrations. Moreover, the absence of time in these equations precludes any change from occurring during the reaction course. These are serious objections, but let us explain what the limitations of this work are and are not.

Hill’s terse style gives additional importance to each sentence in his book [7]. The first sentence of the preface draws the reader’s attention to the book’s subtitle: “The Steady-State Kinetic and Thermodynamic Formalism”. The steady-state approximation that we used in all calculations, as Hill did, does not allow for changes with time. We assumed constant fixed concentrations to ensure the non-equilibrium situation—that is, the chemiostatic condition when concentrations are kept steady by continuous product withdrawal and substrate injection. The substrate concentration did not disappear from the system after the multiplication by the first forward second-order rate constant. The product concentration did not disappear after the multiplication by the reverse second-order binding rate constant. Thus, the concentration dependence survived when we followed Hill in treating all transitions between functionally important enzyme states as first-order processes.

For each reaction, we used the substrate concentration and temperature mentioned in the corresponding reference (see the Supplementary Materials), where we found observed or estimated kinetic constants. An element of subjectivity remained when we could not find the calculated, stated, or physiological product concentration for the reaction that the research paper of interest studied. Product concentrations were often variable in the cited literature. We aimed to keep X/(RT) positive for each reaction, roughly from 1.0 to 10.0. Choosing a 10 times lower product from substrate concentration achieved that goal for about half of the explored reactions. For the remaining reactions, the product concentration had to range from 25 times higher to 1000-fold lower than the substrate concentration to achieve the same goal. 

In rare cases, one of the transitions was irreversible, essentially going only in the forward direction. This was the case for the chorismate mutase (AROH), with its reverse rate constant in the second transition estimated as k_4_ = 10^−8^ (s^−1^) [77]. A high X/(RT) ≈ 23 force could not be avoided (see the Supplementary Materials). We also included *Escherichia coli* β-galactosidase (GAL) data due to its importance as a test case on how single enzyme molecules work. However, the reverse binding constant k_4_ for the product is close to zero but unknown for that enzyme [40,99]. Setting the reverse rate constant equal to zero for the second transition would prevent the calculation of entropy production. The choice of some small number different from zero is then, to some degree, arbitrary. When we used the choice k_4_ = 10^−5^ s^−1^ [99], we obtained the same result of 2553 s^−1^ for overall dissipation as these authors, a satisfactory confirmation that Hill’s method leads to the identical result for overall entropy production when a different method is used to describe the same non-equilibrium steady state. On the other hand, the corresponding affinity X/(RT) = 16.8 is on the high side. In Dataset S1 and Table 1, we chose 10 times lower product concentration (from substrate concentration) and k_4_ = 0.1 s^−1^. That choice led to the overall dissipation/(RT) = 1153.5 s^−1^ and the thermodynamic force X/(RT) = 7.6. In each case we verified the invariance in the quotient between second-order and first-order kinetic constants in each direction, giving the same equilibrium constant from the J = 0 requirement or Haldane relationship.

One can explore whether the logarithmic proportionality between dissipation and kinetic parameters survives when the system relaxes toward equilibrium. That question is beyond the scope of the present paper. Interested readers can study our previous publication [19] for the regularities during the catalysis time course for β-galactosidase (GAL), glucose isomerase (GI), three β-lactamases (Lac1, RTEM, and PC1), ketosteroid isomerase (KSI), triosephosphate isomerase (TPI), and three carbonic anhydrases (CAII, CAII-T200H, and CAI). These are 10 among the 58 enzymatic reactions that we included here only as a snapshot of steady states. To conclude this section, the results presented in Figure 2 are unexpected and essential; they connect fundamental physics and key kinetic parameters in biochemistry, highlighting that high catalytic efficiency does not necessarily require low or minimal dissipation.

### 4.2. Why Must Performance Gain Be Paid with Higher Dissipation?

We argued in a previous text about the unexpected alignment of dissipation with the turnover number and enzyme efficiency. Although surprising, the correlations do exist. Do we know why something known as a useless outcome (that cannot perform work) is tied to obviously beneficial performance gains? The turnover number k_cat_ is an idealized maximal product release rate. While it always contributes to entropy production during enzyme cycling, its contribution depends on the reversibility of enzyme action in a dissipative environment. For instance, prolyl cis ↔ trans interconversion is a reversible process catalyzed by the cyclophilin family members GeoCyp, CypA, CypB, and CypC [78]. Figure 2A illustrates a near-perfect straight-line arrangement of (k_cat_, ϕ/RT) red points for these isomerases with coordinates (37, 92), (97, 238), (103, 246), and (115, 270) (see Table 1). Similar k_cat_ and net flux J values for each isomerase and almost identical X/RT values (see Table 1) explain such a good alignment of dissipation and the catalytic rate k_cat_. The fold improvements shown in Table 2 for cyclophilins k_cat_ are impressive, confirming that k_cat_ is governed by rate-limiting chemistry in the product release step. The calculation of the free-energy profile for CypA and GeoCyp can be performed using the data provided in our Dataset S1 for a nice illustration of how k_cat_, presented as a vertical vector, can strongly influence the rate-limiting barrier height, partial entropy production associated with the product release transition, and overall dissipation. The profile should look similar to Figure 6A from Johnson’s publication [50]. A free-energy profile can be obtained from our data for all 58 reactions, but that task is beyond the scope of this paper. Computational and NMR studies are essential for a better understanding how proteins’ dissipative dynamics enhance the reaction rates in cyclophilins and other enzymes [36,100].

The enzymes reduce the activation energy required to overcome a barrier to the reaction progress in converting substrate to product. The product release and high turnover number would not be possible without prior productive substrate capture. In an older, insightful paper, Northrop proposed [101] that evolution favors increasing both capture and release in a constant ratio. Enzymes’ efficiency depends on their ability to lower energy barriers for and after substrate binding in the reaction energy diagram. The k_cat_/K_m_ can be regarded as a capture constant with a visual interpretation in the activation energy diagram [50,101,102]. In some of the cases that we studied, the enzyme specificity constant k_cat_/K_m_ governs the height of the highest barrier in the free-energy profile (see [102] and Figure 6C from Johnson’s publication [50]). Lowering the activation energy barriers increases the net flux J in the forward direction even when high positive affinity does not change. Thus, the overall dissipation must also increase. We can verify from the data in Table 1 and Figure 2B that the (k_cat_/K_m_, ϕ/RT) points are close to the regression line for X/RT values around 10 and higher than 10.

### 4.3. Does the Sublinear Scaling Law for Enzymes Point Toward the Origin of Kleiber’s Law?

Figure 2B shows that the dissipation rate of enzymes, expressed as dissipation/RT, is proportional to their catalytic efficiency raised to a power of 0.73. This finding resembles the scaling law proposed by Max Kleiber nearly a century ago [59,103]. Kleiber concluded that “the mean, standard metabolic rate of mammals amounts to 70 times the 3/4 power of their body weight [103]”. The similarity between the exponents (0.73 and 0.75) may be purely coincidental. Furthermore, other scientists have rediscovered scaling laws in species and ecological systems not considered by Max Kleiber, raising questions about the universality of Kleiber’s law [104,105,106]. The superlinear scaling in prokaryotes [107] further challenges the notion of a universal allometric dependence with the exponent 2/3 or 3/4 [108].

The question of the origin of Kleiber’s law has a complex history [109,110]. Enzymes are crucial because neither body mass nor the associated metabolic rate would be possible without their activity. We can consider dissipation as a substitute for metabolic rate, while enzyme efficiency serves as the fundamental cause of organic mass accumulation. For example, the efficient enzymes found in *E. coli* bacteria facilitate rapid nutrient assimilation and conversion into biomass, promoting fast growth in nutrient-rich environments. However, in animals, bioenergetics and body heat dissipation processes are primarily driven by the work of mitochondrial membrane enzymes [111] rather than the mostly soluble enzymes discussed in this paper. Establishing a scaling law between dissipation and the effective accumulation of organic compounds during respiration or photosynthesis remains challenging, mainly due to the more complex kinetics of membrane enzymes.

Still, for the present database of 58 reactions, statistical analysis indicated the dissipation and the turnover number k_cat_ scales in a nearly linear manner, while the dissipation and enzyme efficiency k_cat_/K_m_ scale as the power-law sublinear function y = 0.015∙x^0.73^ (Figure 2B). The robustness of the exponent estimate at 0.73 for the dependence of dissipation on enzyme efficiency stems from a reasonably narrow 95% confidence interval (0.67–0.80) after re-sampling the data. The proposed sublinear scaling law between dissipation and the catalytic efficiency of enzymes encompasses Bacteria and Archaea as the majority and the Eukaryota domain as a minority among the considered species (see Dataset S1 in the Supplementary Materials). Thus, discovering a unifying principle in metabolism already at the level of enzymes is a challenge that this contribution initiated for the case of soluble enzymes with a most straightforward uni-uni catalytic mechanism.

### 4.4. Did We Gain Additional Insight into the Proposal That Specialized Enzymes Evolved from Primitive Generalist Enzymes?

The next question for discussion is the relevance of the presented results to Jensen’s proposal [46], which states that specialized enzymes evolved from primitive generalist enzymes. Jensen and most other authors exploring his hypothesis considered biological evolution to be separate from universal thermodynamic evolution. In this paper, we have adopted the perspective that biological evolution harnessed, regulated, and enhanced thermodynamic evolution whenever possible to create new dissipation channels and flows. Only one generalist enzyme exists among the 30 enzyme-catalyzed reactions with the highest dissipation (Dataset S1). In contrast, 7 of the 28 low-dissipation reactions involve bifunctional or generalist enzymes. A statistically significant likelihood is that specialized enzymes are associated with higher dissipation rates. Notably, primitive generalist enzymes do not exist anywhere in the current biosphere. Most enzymes in present-day cells are specialized, with a smaller proportion being promiscuous or multifunctional. In conclusion, our findings (Table 1, Figure 2, and Dataset S1 in the Supplementary Materials) indicate that higher dissipation rates distinguish specialized from generalist enzymes.

The specific examples of generalist and specialized variants derived from the same enzyme provide a basis for testing the proposed physical extension of Jensen’s hypothesis. To investigate this, we compared the dissipation allocation between specialized and generalist kynureninase variants [47]. The specialized mutant exhibited approximately twice the total dissipation compared to the generalist mutant enzyme (see Figure 3). Even after adjusting the concentrations to create the same driving force for both mutants, the specialized variant still showed higher dissipation (see the Supplementary Materials). Additionally, we observed a consistent trend of increased dissipation and improved performance parameters in evolutionarily related specialized enzymes, as illustrated by the more advanced β-lactamase enzymes (see Figure 4 and Figure 5). This increase in dissipation and efficiency may be typical of specialized orthologous enzymes with greater evolutionary distance from a common ancestor. Similarly, for *Escherichia coli* β-galactosidase, other researchers [99] concluded that a more efficient enzyme is associated with higher total dissipation. However, their study only examined three combinations of microscopic rate constants within a two-state kinetic model. A reader skilled in phylogenetic analysis could explore the relationships (evolutionary distances) among the cyclophilin family members CypA, CypB, CypC, and GeoCyp (see [78]). All other data for finding possible trends with dissipation and performance parameters are already in Table 1.

### 4.5. Simulating the Effect of Stochastic Noise

We generated substantial additional synthetic data by simulating the impact of stochastic noise on microscopic rate constants. When we uniformly accelerated the process for all catalytic steps (forward variations), we observed nearly perfect proportionality between kinetic performance and dissipation across all reactions (see the Supplementary Materials). Under the same fixed force constraint, while allowing for variations in compensatory substrate–enzyme associations and enzyme–product dissociations (trade-off variations), we found that enzyme efficiency exceeded the experimental observations for 41% of reactions, and the catalytic constant was higher for 59% of reactions. However, in our dataset of 58 enzyme-catalyzed reactions, there was no case where our maximum dissipation requirement improved both optimal enzyme performance parameters: k_cat_ and k_cat_/K_m_.

Figure 3A,C present examples of simulations for kynureninase variants [47], referred to as “forward variations”. We also utilized these variants to demonstrate the impact of maximum entropy production requirements on potential steady states, known as trade-off simulations (see Figure 3B,D). The generalist mutant exhibits a higher potential for evolvability regarding catalytic efficiency, as shown in Figure 3A,B. Specifically, these simulations help identify significantly higher optimal enzyme efficiency (as illustrated in Figure 3B for the generalist mutant) or slightly lower optimal enzyme efficiency (as shown in Figure 3D for the specialized variant) than the observed values. Both scenarios are associated with a slight increase in dissipation relative to the observed measurements.

The theoretical possibility of improving enzyme performance parameters by increasing overall dissipation requires experimental verification. There were no attempts to direct the evolution of enzymes toward higher or maximal dissipation. Laboratory-evolved enzymes [112] and coupled enzyme systems can incorporate hard-to-predict mutations and novel functions by artificial selection for higher dissipation, as measured by microcalorimetry. Table 2 enumerates predicted fold changes (regarding observed values) in the turnover number and enzyme efficiency in trade-off simulations for the maximum dissipation.

### 4.6. Comparison with Earlier Simulations

Earlier simulations, called “fixed-forward-product” simulations, were designed to find the maximum total entropy production in cyclic enzyme catalysis and operated under different constraints [113,114]. One key requirement of these simulations was that the product of all forward kinetic constants k_i_ was fixed to its observed value. This approach also led to maximal ϕ and optimal performance parameters. However, the maximal ϕ and optimal parameters derived from these simulations can often be significantly smaller than the dissipation, k_cat_, and k_cat_/K_m_ values calculated using the observed values for the microscopic kinetic constants (see references [16,19] for examples using PC1 β-lactamase parameters).

Typically, the fixed-forward-product simulations result in an unrealistic distribution of state probabilities across each conformational state and an equal allocation of dissipation among each conformational transition. However, the partial entropy productions associated with transitions between enzyme functional states are unequal when the goal is flux maximization [55]. Forcing the enzyme to have a similar steady-state probability for all functional states and an equal dissipation allocation to each catalytic step can destroy its biological function [114].

Each transition can be optimized using our theorem for maximal partial entropy production [17,18]. We introduced maximal-partial-dissipation simulations, which have a significant advantage: our research over the past 20 years has shown that these simulations yield higher-than-observed performance parameters and total dissipation in each enzyme-catalyzed reaction that we examined. Examples can be found in reference [17]. Verification for the 58 reactions discussed here is beyond the scope of this contribution.

### 4.7. The Support for the “Evolution-Coupling Hypothesis”

To connect all of the threads, we can explore the unforeseen benefits that arose from our focus on dissipative free-energy channeling involving 51 enzymes from various kingdoms of life. These enzymes enable downstream processes that partially convert free energy into biological power. Although these enzymes do not collectively form any known metabolic network, we have identified an unexpected link between catalytic performance parameters and their associated dissipation. This connection stems from the enzymes’ ability to open specific dissipation channels.

We propose that these enzymes are products of both biological evolution and universal thermodynamic evolution. They occupy an essential position in the hierarchy of free-energy harvesting, channeling energy to meet cellular needs, and releasing dissipation to the environment to maintain temporary homeostasis and the necessary time lag for realizing growth potential. Their activity accelerates the thermodynamic evolution of the environment.

As biological evolution has advanced over the ages, it has favored the emergence of increasingly complex organisms that could retain captured free energy for longer durations [30]. Extending the time lag for reproduction did not inhibit the increase of entropy production in the environment beyond what would occur without mature species. For instance, our Table 1 and Dataset S1 include examples of mammalian enzymes that are as proficient at dissipating energy as the best bacterial enzymes. Furthermore, cases of enzymes showing modest or low performance and dissipation highlight the significant roles of regulation and dynamic kinetic stability in understanding biological evolution, provided that thermodynamic constraints are met [115,116].

The evolution of chemistry to life may occur in favorable circumstances beyond Earth, but not without being powered by the dissipation of available gradients [117]. For instance, harnessing and dissipating the geochemical chemiosmotic potential is how life may have originated at submarine hydrothermal vents [118]. The present contribution supports the “evolution-coupling” hypothesis, which posits a link between thermodynamic and biological evolution [14,119].

The synergy between thermodynamic and biological evolution is one possible explanation for why enzyme performance parameters are related to overall dissipation. It is attractive because of nature’s spontaneous tendency to dissipate force gradients whenever barriers decrease or vanish for corresponding flows. The cyclical activity is essential to most enzymes, as reflected in the name of the turnover number. It takes place at room temperature in a highly dissipative environment. Various biological evolutionary pressures can influence enzyme performance but cannot eliminate dissipation, except in the quantum realm. If thermodynamic evolution does not exist, that would eliminate the evolution-coupling hypothesis, but not dissipation due to the movements of electrons, protons, atoms, water molecules, and protein loops within the protein microenvironment. Such movements are essential for conformational and chemical changes after substrate capture. They are not random and cannot be divorced from evolutionary advances. Specifically, we propose that the increase in coupled catalytic efficiency and entropy production is fundamental to the evolution of enzymes. A broader perspective highlights the crucial role of dissipating available free-energy gradients in the least time as both a cause and a consequence of life.

## 5. Conclusions

Enzymes play a crucial role in the metabolism of all living cells. Internal entropy production and exported dissipation are key indicators of enzymatic catalytic activity. Calculating the steady-state entropy production for any enzyme is straightforward if all of the relevant microscopic rate constants and concentrations are known. We compiled a database of enzymes that includes important kinetic and thermodynamic performance parameters, and we examined their relationships. Our research revealed that the performance of enzymes increases alongside their dissipation. The most advanced forms —often called “perfect” or “highly specialized” enzymes—are associated with the highest dissipation levels. These findings suggest a synergy between thermodynamic and biological evolution, highlighting its significance in biology, especially when living systems are not artificially isolated from their environments.

## Figures and Tables

**Table 1:** Catalytic efficiency, catalytic constant, net flux, force, and overall dissipation for 58 enzyme-catalyzed reactions. Reactions are ordered with the symbol “#” from the most to the least dissipation. The correlation among k_cat_/K_m_ and ϕ/(RT) values (highlighted numbers) is the central result of this paper. We refer the reader to the Supplementary Materials for additional details.

#	Enzyme ^&^	k_cat_/K_m_ (Ms)^−1^	k_cat_ (s^−1^)	J (s^−1^)	X/(RT)	ϕ/(RT) (s^−1^)	#	Enzyme	k_cat_/K_m_ (Ms)^−1^	k_cat_ (s^−1^)	J (s^−1^)	X/(RT)	ϕ/(RT) (s^−1^)
1	CAII	83 , 600 , 000	805,433	421,874	1.81	125 , 000	30	GeoCyp	847,000	36.85	33.75	2.73	92.19
2	KSI	302,000,000	35,031	13,756	8.43	115,900	31	ALaO	295,908	6.8	6.58	9.83	64.72
3	CAII-T200H	67,700,000	209,682	33,593	1.87	62,920	32	EpiT	10,333	341	28	1.03	28.9
4	CAI	24,810,000	77,746	15,691	1.81	28,370	33	NSAAR	2857	20	10.1	2.34	23.67
5	Lac1	26,030,200	1905	1757	8.27	14,526	34	iPGM	104,762	22	8.75	2.32	20.29
6	RTEM	23,513,000	975	873	7.74	6757	35	ALiO	8890	1.602	1.276	10.01	12.77
7	sgPPase	70,427,239	812	625	9.95	6214	36	API	50,333	100	10.7	1.08	11.58
8	GPI *	21,721,831	1550	855	3.42	2928	37	RPI	15,143	33.3	5.16	2.12	10.93
9	GAL	1,918,000	730	152	7.6	1154	38	RacE2mut	14,081	81.67	4.36	2.24	9.76
10	coliMgPPase	44,481,928	147	128	8.71	1116	39	TIProRC *	1996	12.13	1.64	3.54	5.82
11	yeastPPase	29,765,749	189	122	7.74	946	40	LYSROEN	318	3.5	2.39	2.4	5.73
12	MR	1,080,105	632	317	2.52	798	41	TIProR *	2300	2.783	1.989	2.26	4.485
13	PC1	10,100,000	60.8	60.6	11.37	689	42	KYNase_66	34,526	0.74	0.602	7.03	4.233
14	FH	6,355,555	1833	508	1.35	687	43	RacE2	5311	32.4	1.39	2.84	3.958
15	KSI-D38E	2,769,512	129	73	8.97	655	44	ATAmut2	1427	1.87	0.986	2.07	2.037
16	KIN	2,225,553	106	41.4	13.73	569	45	KYNase_93D9 *	38,565	0.67	0.424	4.77	2.022
17	RPE	1,605,047	305	191	2.91	554	46	ATAmut1	4875	1.95	1.123	1.59	1.785
18	dPGM	1,650,000	330	179	2.37	423	47	TM0831 *	17.9	2.156	0.244	2.53	0.618
19	PMI	595,000	800	161	2.27	365	48	ATA	217	0.5	0.216	2.06	0.444
20	AROH	589,474	50.4	12.8	22.59	289	49	FAProR *	18.9	0.597	0.164	1.32	0.216
21	CypC	471,129	115	99.6	2.71	270	50	HcmABwt	481	0.05	0.048	3.43	0.163
22	ALF	5,086,315	52.6	51.8	5.14	266	51	SerR	31	0.31	0.056	2.62	0.148
23	CypB	379,186	103	90.1	2.73	246	52	TAM	37.8	0.0209	0.0135	8.47	0.114
24	EpiI	100,400	502	92.6	2.65	245	53	HcmAmut	10.9	0.02	0.0165	4.05	0.0666
25	TPI	542,518	714	209	1.16	242	54	NSAARN	3.89	0.07	0.023	2.86	0.0659
26	ALS	3,255,879	40.2	39.4	6.05	239	55	EpiTmut	15.9	4.9	0.0474	0.94	0.044
27	CypA	936,569	97.3	87.0	2.73	238	56	GI *	0.0365	0.029	0.0097	2.36	0.023
28	AR	172,197	1692	827	1.80	229	57	HcmIcm	1.8182	0.001	0.00094	5.21	0.0049
29	yPGM	747,211	380	93.4	1.03	96.2	58	GI3 *	0.00018	0.00012	0.000015	2.96	0.000044

* Generalist or bifunctional enzymes. All others are specialized enzymes. ^&^ Abbreviations: CAII = carbonic anhydrase II, KSI = ketosteroid isomerase, CAII-T200H = T200H mutant of carbonic anhydrase II, CAI = carbonic anhydrase I, Lac1 = β-lactamase Lac1, RTEM = β-lactamase RTEM, sgPPase = soluble inorganic pyrophosphatase, GPI = glucose-6-phosphate isomerase, GAL = β-galactosidase, coliMgPPase = soluble inorganic pyrophosphatase with Mg^2+^, yeastPPase = yeast soluble inorganic pyrophosphatase, MR = mandelate racemase, PC1 = β-lactamase PC1, FH = fumarate hydratase, KSI-D38E = D38E mutant of ketosteroid isomerase, KIN = kinesin-1, RPE = ribulose-5-phosphate epimerase, dPGM = phosphoglycerate mutase dPGM, PMI = mannose 6-phosphate isomerase, AROH = chorismate mutase, CypC = cyclophilin C, ALF = fructose 1,6-bisphosphate aldolase, CypB = cyclophilin B, EpiI = isoleucine 2-epimerase, TPI = triosephosphate isomerase, ALS = sedoheptulose 1,7-biphosphate aldolase, CypA = cyclophilin A, AR = alanine racemase, yPGM = phosphoglycerate mutase, GeoCyp = cyclophilin GeoCyp, ALaO = altro-octulose 1,8-bisphosphate aldolase, EpiT = D-psicose 3-epimerase, NSAAR = n-succinyl amino acid racemase, iPGM = phosphoglycerate mutase iPGM, ALiO = arabinose-5-phosphate aldolase, API = arabinose-5-phosphate isomerase, RPI = ribose-5-phosphate isomerase, RacE2mut = glutamate racemase mutant R25A, TIProRC = proline racemase from the archaeon T. litoralis, LYSROEN = lysine racemase, TIProR = proline racemase from the archaeon T. litoralis with L-proline substrate, KYNase_66 = kynureininase mutant 2, RacE2 = glutamate racemase, ATAmut2 = (R)-selective amine transaminase mutant 2, KYNase_93D9 = kynureininase mutant 1, ATAmut1 = (R)-selective amine transaminase mutant 1, TM0831 = TM0831 racemase, ATA = (R)-selective amine transaminase, FAProR = proline racemase from the archaeon F. acidiphilum, HcmABwt = 2-hydroxyisobutyryl-CoA-mutase, SerR = serine racemase, TAM = tyrosine aminomutase, HcmAmut = 2-hydroxyisobutyryl-CoA-mutase mutant I90V, NSAARN = n-succinyl amino acid racemase acting on L-acetyl-D-asparagine substrate, EpiTmut = D-psicose 3-epimerase mutant R215K, GI = glucose isomerase Sweetzyme T^®^, HcmIcm = 2-hydroxyisobutyryl-CoA-mutase acting on isobutyryl-CoA, GI3 = glucose isomerase Sweetzyme IT^®^.

**Table 2:** Fold changes (regarding Table 1 values) in optimized k_cat_ and k_cat_/K_m_ for maximal dissipation that we obtained in trade-off simulations.

#	Enzyme ^&^	k_cat_/K_m_ Fold	k_cat_ Fold	J (s^−1^)	X/(RT)	ϕ/(RT) max (s^−1^)	#	Enzyme	k_cat_/K_m_ Fold	k_cat_ Fold	J (s^−1^)	X/(RT)	ϕ/(RT) max (s^−1^)
1	CAII	2.85	0.85	516,052	4.12	2.1 × 10^6^	30	GeoCyp	0.13	21.9	362	2.73	989
2	KSI	2.23	0.78	15,963	8.43	134,498	31	ALaO	0.34	1.07	6.86	9.83	67.5
3	CAII-T200H	3.77	0.67	45,786	1.87	85,759	32	EpiT	1.20	0.60	29.6	1.03	30.6
4	CAI	1.98	0.63	18,986	1.81	34,328	33	NSAAR	0.74	1.75	11.2	2.34	26.3
5	Lac1	0.33	1.56	2364	8.27	18,719	34	iPGM	0.89	1.17	8.84	2.32	20.5
6	RTEM	0.21	2.05	1365	7.74	10,570	35	ALiO	1.98	0.98	1.30	10.01	13.0
7	sgPPase	0.95	1.02	625	9.95	6218	36	API	1.08	0.78	10.9	1.08	11.7
8	GPI *	0.75	1.60	931	3.42	3185	37	RPI	1.44	0.58	5.73	2.12	12.1
9	GAL	0.94	2.62	165	7.60	1253	38	RacE2mut	3.18	0.29	6.54	2.24	14.6
10	coliMgPPase	0.44	1.31	145	8.71	1263	39	TIProRC *	1.68	0.47	2.01	3.54	7.14
11	yeastPPase	0.56	1.30	131	7.74	1013	40	LYSROEN	0.49	2.86	3.50	2.40	8.41
12	MR	1.40	0.88	329	2.52	829	41	TIProR *	0.39	3.64	3.54	2.26	7.98
13	PC1	0.06	2.55	60.6	11.37	1519	42	KYNase_66	0.64	1.23	0.637	7.03	4.48
14	FH	0.88	1.29	513	1.35	694	43	RacE2	3.45	0.28	1.94	2.84	5.50
15	KSI-D38E	3.41	1.00	126	11.97	1507	44	ATAmut2	0.67	1.93	1.15	2.07	2.37
16	KIN	1.30	0.85	42.3	13.73	580	45	KYNase_93D9 *	1.86	0.95	0.438	4.77	2.09
17	RPE	0.66	2.27	240	2.91	697	46	ATAmut1	0.43	3.55	1.77	1.59	2.82
18	dPGM	0.68	1.77	201	2.37	477	47	TM0831 *	2.17	0.42	0.317	2.53	0.804
19	PMI	1.13	0.77	164	2.27	372	48	ATA	0.77	1.66	0.231	2.06	0.475
20	AROH	2.47	0.71	15.1	22.59	341	49	FAProR *	0.56	1.93	0.180	1.32	0.237
21	CypC	0.22	2.43	188	2.71	509	50	HcmABwt	0.19	22.0	0.449	3.43	1.54
22	ALF	0.06	16.4	520	5.14	2671	51	SerR	1.28	0.65	0.060	2.62	0.157
23	CypB	0.26	3.43	214	2.73	584	52	TAM	3.75	1.00	0.0208	10.08	0.210
24	EpiI	1.06	0.85	93.2	2.65	247	53	HcmAmut	0.49	4.00	0.0302	4.05	0.122
25	TPI	0.88	1.39	218	1.16	253	54	NSAARN	0.99	1.01	0.023	2.86	0.066
26	ALS	0.10	2.50	84.5	6.05	512	55	EpiTmut	1.13	0.35	0.0516	0.94	0.048
27	CypA	0.15	8.35	407	2.73	1110	56	GI *	0.98	1.03	0.0097	2.36	0.023
28	AR	3.68	0.91	977	1.79	1751	57	HcmIcm	0.35	9.00	0.0037	5.21	0.0192
29	yPGM	0.88	1.32	95.5	1.03	98.4	58	GI3 *	2.49	0.48	0.000024	2.96	0.00007

See Table 1 for the meaning of the #, * and ^&^ symbols. Underlined numbers for CAII, KSI-D38E, AR, and TAM are cases where we had to increase the ligand concentrations to find the maximum dissipation. The performance parameters highlighted in gray distinguish enzymes for which trade-off variations can find higher enzyme efficiency than observed (Eff-enzymes) from those with the potential for a higher turnover number (Tur-enzymes).

## Data Availability

The data presented in this study are openly available in https://github.com/DJureticSplit/PERF-ENZYMES. (accessed on 27 March 2025).

## Supplementary Materials

The following supporting information can be downloaded at https://www.mdpi.com/article/10.3390/e27040365/s1. Dataset S1: names of corresponding source codes for simulating each of the 58 enzyme-catalyzed reactions and the data needed to construct figures.
