# Supplementary material for: Exploring the Evolution-Coupling Hypothesis: Do Enzymes’ Performance Gains Correlate with Increased Dissipation?
Source: Entropy (Basel). 2025 Mar 29;27(4):365. doi: 10.3390/e27040365 (PMC12025749; doi:10.3390/e27040365)
Supplement: Supplementary file 1 [file entropy-27-00365-s001.zip › Juretic-Supplementary-Materials-and-Source-Codes.pdf]

Supplementary Materials, Data Deposit and Software Source Codes for:

Exploring the Evolution-Coupling Hypothesis: Do Enzymes' Performance Gains Correlate with Increased Dissipation?

Davor Juretić*

Faculty of Science, University of Split, Ruđera Boškovića 33, 21000 Split, Croatia

*Correspondence: davor.juretic@gmail.com

The SM files are:

Extended Methods (below)
Dataset S1 (enclosed Excel file)
Source codes for FORTRAN programs

The essential supporting dataset (Dataset S1 Excel file) contains names of corresponding source codes for simulating each of the 58 enzyme-catalyzed reactions and the data needed to construct figures. All of our 58 FORTRAN programs (see column Y for their names) for finding parameters from experimental data and their optimal or maximal values are available on GitHub (https://github.com/DJureticSplit/PERF-ENZYMES). The Dataset S1 Excel file is also freely available for download at the same link. Although enzyme performance parameters and corresponding dissipation values are in Table 1 from the main text, we suggest that manuscript readers also consult the complete bioinformatics data from Dataset S1. For a chosen reaction, the column (AA) from the Dataset S1 Excel file has the reference from which we extracted observed or estimated data. A total of 30 references for research papers have the same associated number in the column AC as in the main text.

The input data for source codes are available in the supporting dataset (columns J, K, L, M, N, O, P, Q, R, S, T, U, V, and Z). Besides enzyme and substrate names, these are numbers of conformational states, concentrations of substrates and products, forward rate constants (with odd index), and backward rate constants (with even index). In the case of second-order processes, second-order rate constants are multiplied with the concentration of ligand to obtain first-order rate constants for all transitions (expressed in inverse seconds). The odd-even pair of rate constants 1-2, 3-4, 5-6, and 7-8 belong to the conformational transitions „i = 1, 2, 3, 4," and their ratio is the equilibrium constants K_i. Each Excel (SM) dataset row with numbers in columns B and C contains x and y coordinates for one Figure 2a point. Each row with numbers in columns A and C contains x and y coordinates for one Figure 2b point.

We assumed that the system could jump among quasi-steady states under the conditions of fixed temperature and positive driving force. The output of each program

contains from 1000 to 30000 rows. All data from the first row filled with numbers from the output file in the ".dat" format are published parameters from papers listed in the SM dataset, and additional parameters we calculated from the published data. The remaining rows in the output ".dat" file reflect the variation type we introduced by multiplication of k_i constants with stochastic noise (forward or trade-off variations). For instance, the HsKYNase versions from columns J and Y (rows # 43 and # 46) are the enzyme and FORTRAN source code titles needed to construct Figure 3, panels b and d, when using the trade-off simulations. Representative source codes for human kynureninase variants are HsKYNase-66-SIout3 and HsKYNase-93D9-SIout3. Their outputs are data files for a) Dataset S1 values (rows # 43 and # 46 values), b) for forward variations, and c) for trade-off variations. The b) and c) output "dat" files contain the Dataset S1 parameters in their first row with numbers. The same condition that X/RT = 7.3 for programs using parameters from both variants (named HsKYNase93D9-SameX.for and HsKYNase66-SameX.for) served to check if the dissipation differences survive in the output parameters for the same driving force X/RT = 7.3. In that case, the calculated entropy production is higher for the specialized mutant 66, while an optimal catalytic efficiency is higher for the generalist mutant 93D9, just as representative source codes revealed.

The conversion of the "dat" format file into an Excel file facilitates finding optimal parameters for maximal overall dissipation in the case of the trade-off simulations. The rows are then easily ordered from the maximal to minimal dissipation. To facilitate reproduction, we added the kinesin-1 "dat" example and its conversion into an Excel output file from the FORTRAN program KIN-k1k4k1k7.for.

We sometimes increased the substrate and product concentration to find the maximum dissipation. That was the case with alanine racemase (AR). Increasing substrate and product concentration 20 times while keeping the same 10:1 ratio in favor of substrate concentration was enough to find the maximal dissipation and optimal performance parameters. The other case was carbonic anhydrase II (CAII). We had to increase the substrate concentration 10 times to find the dissipation maximum. The third and fourth cases were the tyrosine aminomutase (TAM) and D38E mutant of ketosteroid isomerase (KSI-D38E). For the reproduction of these results, we included FORTRAN source codes AR-K1k4k1k7.for, ARforSI-K1K4k1k7.for, ARmaxEP-K1K4k1k7.for, CAII-K1K4k1k7.for, CAIImax-K1K4k1k7.for, KSI-D38E-K1K4k1k7.for, KSID38EmaxEP-K1K4k1k7.for, TAM-K1K4k1k7.for, and TAMmaxER-K1K4k1k7.for in our package of source codes.

The source codes are more straightforward for forward variations after introducing stochastic noise. Only minimal changes are needed when new enzyme reactions are simulated using such variations. We enclosed FORTRAN programs named KSI-forw.for, CAI-forw.for, CAII-forw.for, CAIIT200H-forw.for, HsKYNase-66-forw.for, and HsKYNase-93D9-forw.for) for performing forward variations (with the "forw" in their title) to facilitate the construction of analogous source codes for other enzymes of interest to readers. When the enzyme of interest is already included in Dataset S1, it is sufficient to take relevant kinetic data from Dataset S1 and insert them into corresponding source codes.

In several cases, we verified the invariance in the quotient between second-order and first-order kinetic constants in each direction, giving the same equilibrium constant from the J = 0 requirement or Haldane relationship. Obtained equilibrium constants from such calculations agree with reported values for corresponding enzymes. We used the following source codes for these verifications: GALcoliX-K1K2k1k3.for, GALcoli-K1K2k1k3.for, GPIhrbc-K1K2k1k3.for, and TPIyeast-K1K4k1k7.for.

The source codes for constructing Figure 5 are PC1-toff.for, RTEM-toff.for, and Lac1-toff.for. The first row with numbers from the output ".dat" file is enough to build the panels a, b, and c from that figure when combined with earlier results about evolutionary distances (see Figure 4 legend). We extracted the dissipation maximum and corresponding optimal kinetic parameters from the remaining rows for the trade-off variations. Beta-lactamases PC1, TREM, and Lac-1 are "Tur-type" enzmes (see the main text). Thus, we examined in Figure 5d the relationship among optimal turnover numbers kcat and evolutionary distances from putative common ancestor enzyme.
